# Supplementary material for: The Population-Wide Risk-Benefit Profile of Extending the Primary COVID-19 Vaccine Course Compared with an mRNA Booster Dose Program
Source: Vaccines (Basel). 2022 Jan 18;10(2):140. doi: 10.3390/vaccines10020140 (PMC8880242; doi:10.3390/vaccines10020140)
Supplement: Supplementary file 1 [file vaccines-10-00140-s001.zip › vaccines-1530753-supplementary.pdf]

vaccines

The population-wide risk-benefit profile of extending the primary COVID-19 vaccine course compared with an mRNA booster dose program

Supplementary Material

Table of Contents

SUPPLEMENTARY METHODS 2
TRANSMISSION (SEIR) MODEL 2
DISEASE MODEL 3
SUPPLEMENTARY TABLES 5
SUPPLEMENTARY FIGURES 8
REFERENCES 11

List of Tables

Table S1. Population estimates for the UK 5
Table S2. Daily contacts by different age groups in the UK 5
Table S3. Proportions of hospitalised patients, patients in critical care, and deaths, stratified by age group 6
Table S4. Overall myocarditis/pericarditis rates per million doses following mRNA COVID-19 vaccination (all doses) 6
Table S5. The number of COVID-19 hospitalisations prevented per 100,000 primary series or booster vaccines (logarithmic scale) for different levels of vaccination protection waning 7

List of Figures

Figure S1: Quantifying the risk-benefit within the context of a dynamic population 8
Figure S2: Expected daily number of hospitalisations at three months as a function of infection rate, vaccination status and vaccine coverage 9
Figure S3: The number of COVID-19 hospitalisations prevented per 100,000 primary series or booster vaccines (logarithmic scale) for different levels of vaccination protection waning 10

Vaccines **2022**, *10*, 140. https://doi.org/10.3390/vaccines10020140 www.mdpi.com/journal/vaccines

SUPPLEMENTARY METHODS

TRANSMISSION (SEIR) MODEL

We modified a previous transmission model[1] to the following discrete system of nonlinear equations:

$$S_{a,t+1}^{V} = S_{a,t}^{V} - \beta\left(\frac{S_{a,t}^{V}}{N_a}\right)\sum_{j=1}^{k}\frac{M_{a,j}I_{j,t}^{V}}{\tau_I}$$

$$E_{a,t+1}^{V} = E_{a,t}^{V} + \beta\left(\frac{S_{a,t}^{V}}{N_a}\right)\sum_{j=1}^{k}\frac{M_{a,j}I_{j,t}^{V}}{\tau_I} - \frac{E_{a,t}^{V}}{\tau_E}$$

$$I_{a,t+1}^{V} = I_{a,t}^{V} + \frac{E_{a,t}^{V}}{\tau_E} - \frac{I_{a,t}^{V}}{\tau_I}$$

$$R_{a,t+1}^{V} = R_{a,t}^{V} + \frac{I_{a,t}^{V}}{\tau_I}$$

where $S_{a,t}^{V}$ is the susceptible population free from infection, $E_{a,t}^{V}$ is the exposed population but not symptomatic, $I_{a,t}^{V}$ is the symptomatic population and $R_{a,t}^{V}$ is the recovered population. These populations can either be unvaccinated, vaccinated or boosted, represented by the superscript V. The transmission rate is given by the parameter β. The length of time spent in the exposed state is given by τ_E and the length of time spent in the infectious state is given by τ_I.

We adjusted our contact matrix $M_{i,j}$ to a normalized contact matrices whose maximum eigenvalues are equal to 1, similar to Salje et al. 2020[2]. This normalization ensures that estimates of the force of infection are little impacted by the contact matrix but by the effective reproductive number.

We use the relationship between the transmission rate and the reproductive number using[3]

$$\beta = \frac{R_{eff}}{\tau_I \times Maximum\left(Eigenvalue\left(M_{i,j}\right)\right)}$$

where $Maximum\left(Eigenvalue\left(M_{i,j}\right)\right)$ is the maximum eigenvalue of the contact matrix $M_{i,j}$, and R_{eff} is the effective reproductive number.

Vaccine immunity against transmission at time t is assumed to wane linearly at a rate δ according to the equation:

$$\varepsilon(t) = -\delta * t + \varepsilon_0$$

where ε_0 is the initial vaccine efficacy. Different waning rates were explored and how they impact the number of infections and severe cases.

DISEASE MODEL

We assume that hospitalisations (both ward and ICU) and deaths, stratified by vaccination status, i.e., unvaccinated, vaccinated or boosted, are sampled from a Poisson distribution – with the Poisson's rate given as a function the number of new cases. The disease model is given by the statistical model:

$$H_{a,t}^{V} \sim Poisson\left(I_{a,t}^{V} \times \mu_{HOSP,a}\right)$$

$$LC_{a,t}^{V} \sim Poisson\left(\left(R_{a,t}^{V} - R_{a,t-1}^{V}\right) \times \mu_{LCOVID,a}\right)$$

$$ICU_{a,t}^{V} \sim Poisson\left(I_{a,t}^{V} \times \mu_{IICU,a}\right) + Poisson\left(H_{a,t} \times \mu_{HICU,a}\right)$$

$$Dead_{a,t}^{V} \sim Poisson\left(I_{a,t}^{V} \times \mu_{ID,a}\right) + Poisson\left(H_{a,t}^{V} \times \mu_{HD,a}\right) + Poisson\left(ICU_{a,t}^{V} \times \mu_{ICUD,a}\right),$$

where $H_{a,t}$ is the number of new hospital admissions, $LC_{a,t}$ is the number with long COVID, $ICU_{a,t}$ is the number of new patients admitted to the ICU and $Dead_{a,t}$ is the number of new deaths from COVID disease at time t stratified by age a. The parameter $\mu_{HOSP,a}$ is the overall probability of hospitalisation for COVID disease for a subject of age a, $\mu_{LCOVID,a}$ is the probability of developing long COVID, $\mu_{IICU,a}$ is the probability of ICU admission from home, $\mu_{HICU,a}$ is the probability of ICU admission for individuals currently hospitalised in wards, $\mu_{ID,a}$ is the probability of dying of COVID prior to hospitalisation, $\mu_{HD,a}$ is the probability of dying for subjects hospitalised in wards and $\mu_{ICUD,a}$ is the probability of dying for individuals hospitalised in the ICU.

Model parameters and data

Disease transmission was modelled in the UK population using the age distribution of the UK 2019 mid-year populations (Table S1).

The length of time spent by an infected individual exposed to the virus before they become infectious (latent period) was assumed to be 3 days[4]. The length of time spent by an individual in an infectious state was assumed to be 7 days[4, 5]. The probability of ward hospitalizations, ICU admission, long Covid and death are provided in Table S3. Recovered individuals were deemed to be resistant to further infection.

Age-specific population mixing was modelled using published data (Table S2). We assume that mixing patterns have not yet gone back to pre-pandemic levels. The effective reproductive number was fixed at 1.5. The vaccination speed was assumed to be 0.3% and the maximum achievable vaccine coverage was set to 90% across all age categories. All children <10 years remained unvaccinated. Instead of starting the infection with exported cases, we can start the infection process in an setting, with a certain level of infection in the population (incidence was varied between 10 and 600 per 100 000 population). In all scenarios, we assume that vaccination started 12 months ago.

The adverse events due to vaccination considered are myocarditis/pericarditis and the incidence used was taken from the Center for Disease Control' s Advisory Committee on Immunization Practices (ACIP) meeting of June 23 2021[6] (Table S4).

Impact of boosters on hospitalisations

When assessing the impact of boosting on the number of hospitalisation averted over a certain period,

Vaccines 2022, 10, 140. https://doi.org/10.3390/vaccines10020140 www.mdpi.com/journal/vaccines

3 months for illustration purposes, we assumed an infection rate of 400 per 100 000 population at any time point after the primary series.

We generated outcomes for the following scenarios:

- Counterfactual scenario – we assumed that at a given primary series coverage, we stop vaccinating at all and observe the number of severe cases in the next 3 months.
- Primary series with no booster – we assumed that booster is not implemented but coverage of the primary series grows up to 90% in the population and observe the number of severe cases in the next 3 months.
- Primary series with a booster – we assumed that primary series coverage continues to 90% and booster is also introduced at the same rate at which the primary series started (0.3%), and observe the number of severe cases in the next 3 months.

Using these scenarios, we calculated the impact per percentage point of primary series doses given and the impact per percentage point of booster vaccine given on the number of hospitalisations. The number of doses given were calculated using the number of people who were vaccinated in 3 months.

Risk-benefit profile of vaccination

We assess the impact of current vaccine coverage, infection levels on the number of hospitalisations, deaths, long COVID cases and also the number of adverse events (myocarditis/pericarditis) in the vaccinated population during the period of study, i.e., 3 months. Using the incidence of myocarditis (Table S4), we estimated the expected cases of myocarditis by age category and compared that to the number of severe cases observed in the vaccinated population.

SUPPLEMENTARY TABLES

Table S1. Population estimates for the UK

Age category	Population	Proportion (%)
**0-4 years**	3,857,263	5.77%
**5-9 years**	4,149,852	6.21%
**10-14 years**	3,953,866	5.92%
**15-19 years**	3,656,968	5.47%
**20-24 years**	4,153,080	6.22%
**25-29 years**	4,514,249	6.76%
**30-34 years**	4,497,132	6.73%
**35-39 years**	4,395,667	6.58%
**40-44 years**	4,019,539	6.02%
**45-49 years**	4,402,122	6.59%
**50-54 years**	4,661,015	6.98%
**55-59 years**	4,405,908	6.60%
**60-64 years**	3,755,185	5.62%
**65-69 years**	3,368,199	5.04%
**70-74 years**	3,318,867	4.97%
**75-79 years**	2,325,296	3.48%
**80-85 years**	1,715,328	2.57%
**85+ years**	1,647,271	2.47%

Source: Office for National Statistics[7]

Table S2. Daily contacts by different age groups in the UK

Age of contact	Age group of participants														
	00-04	05-09	10-14	15-19	20-24	25-29	30-34	35-39	40-44	45-49	50-54	55-59	60-64	65-69	70+
**00-04**	1.49	0.59	0.25	0.18	0.42	0.61	0.57	0.74	0.18	0.20	0.36	0.15	0.18	0.26	0.07
**05-09**	0.74	3.82	0.53	0.44	0.37	0.42	0.68	0.99	0.66	0.15	0.17	0.19	0.41	0.30	0.07
**10-14**	0.36	0.73	3.19	0.79	0.17	0.14	0.32	0.51	0.69	0.27	0.20	0.19	0.17	0.26	0.17
**15-19**	0.26	0.22	0.52	3.10	0.85	0.12	0.17	0.29	0.48	0.51	0.27	0.13	0.09	0.26	0.37
**20-24**	0.39	0.19	0.09	0.38	1.32	0.49	0.28	0.16	0.23	0.44	0.20	0.28	0.11	0.15	0.00
**25-29**	0.53	0.37	0.11	0.20	0.58	0.64	0.35	0.21	0.23	0.25	0.30	0.30	0.20	0.04	0.03
**30-34**	0.77	0.72	0.29	0.17	0.31	0.42	0.80	0.21	0.27	0.38	0.23	0.20	0.23	0.15	0.07
**35-39**	0.73	0.65	0.61	0.35	0.20	0.24	0.47	0.76	0.47	0.29	0.18	0.11	0.32	0.04	0.03
**40-44**	0.38	0.59	0.58	0.47	0.27	0.29	0.25	0.49	0.45	0.40	0.12	0.19	0.23	0.15	0.30
**45-49**	0.22	0.23	0.22	0.34	0.46	0.29	0.17	0.17	0.35	0.58	0.15	0.19	0.14	0.11	0.20
**50-54**	0.26	0.21	0.14	0.17	0.22	0.17	0.15	0.14	0.19	0.27	0.33	0.35	0.20	0.04	0.07
**55-59**	0.22	0.12	0.10	0.10	0.14	0.22	0.13	0.21	0.05	0.25	0.17	0.52	0.32	0.44	0.10
**60-64**	0.22	0.15	0.10	0.10	0.10	0.14	0.15	0.20	0.19	0.13	0.09	0.24	0.27	0.30	0.13
**65-69**	0.05	0.08	0.07	0.08	0.07	0.12	0.08	0.16	0.11	0.04	0.05	0.07	0.17	0.37	0.43
**70+**	0.09	0.09	0.10	0.08	0.19	0.10	0.05	0.13	0.24	0.29	0.23	0.22	0.11	0.22	0.70

Source: Mossong et al.[8]

Table S3. Proportions of hospitalised patients, patients in critical care, and deaths, stratified by age group

Age group	Proportion of symptomatic cases requiring hospitalisation (%)	Proportion of symptomatic cases who end up dying (%)	Proportion of hospitalised cases requiring critical care (%)	Proportion of critical care patients who end up dying (%)	Proportion with long COVID (%)
**0-9 years**	0.1	0.0001	5.0	40.0	3.1*
**10-19 years**	0.3	0.0003	5.0	40.0	3.1*
**20-29 years**	1.2	0.03	5.0	50.0	3.1
**30-39 years**	3.2	0.08	5.0	50.0	4.7
**40-49 years**	4.9	0.15	6.3	48.6	4.7
**50-59 years**	10.2	0.60	12.2	48.2	3.8
**60-69 years**	16.6	2.2	27.4	48.4	3.8
**70-79 years**	24.3	5.1	43.2	48.6	1.5
**80+ years**	27.3	9.3	70.9	48.0	1.5

Source: Ferguson et al.[9]
Mortality in children taken from Smith et al. 2021[10]
**We assumed the same frequency of long COVID in children and adolescents.*
Source: Opinions and Lifestyle Survey

Table S4. Overall myocarditis/pericarditis rates per million doses following mRNA COVID-19 vaccination (all doses)

Age category	Overall	Females	Males
**12-17 years**	18.1	4.2	32.4
**18-24 years**	15.9	3.6	30.7
**25-29 years**	6.7	2.0	12.2
**30-39 years**	4.2	1.8	6.9
**40-49 years**	2.7	2.0	3.5
**50-64 years**	1.7	1.6	1.9
**65+ years**	1.1	1.1	1.2

Source: Shimabukuro[6]

Table S5. The number of COVID-19 hospitalisations prevented per 100,000 primary series or booster vaccines (logarithmic scale) for different levels of vaccination protection waning

**Waning (per month)**	**Coverage**	**Hospitalisations averted (per 100,000 vaccinations)**	
		**Primary series**	**Booster**
**0.00%**	0.1	46090	5723
	0.2	13627	3825
	0.4	1977	1487
	0.6	302	504
	0.8	31	104
**3.03%**	0.1	44692	5559
	0.2	13487	3819
	0.4	2053	1565
	0.6	319	550
	0.8	29	107
**6.05%**	0.1	42954	5372
	0.2	13168	3763
	0.4	2058	1625
	0.6	327	603
	0.8	32	162
**9.08%**	0.1	40719	5141
	0.2	12608	3674
	0.4	2018	1684
	0.6	325	678
	0.8	36	229
**12.10%**	0.1	37837	4837
	0.2	11781	3545
	0.4	1890	1725
	0.6	314	766
	0.8	39	344
**15.13%**	0.1	34327	4484
	0.2	10624	3361
	0.4	1651	1743
	0.6	284	900
	0.8	38	545

vaccines

SUPPLEMENTARY FIGURES

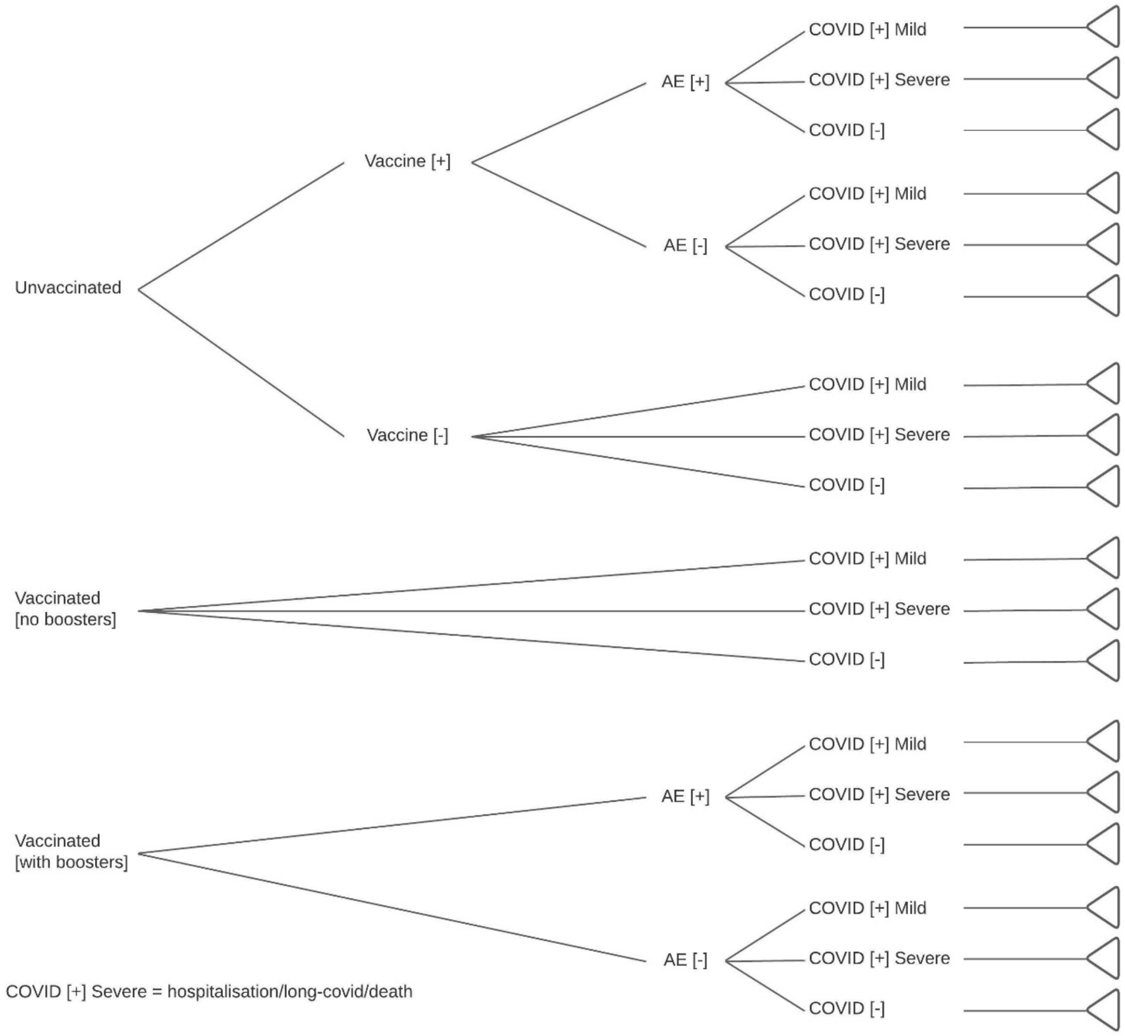

Figure S1: Quantifying the risk-benefit within the context of a dynamic population

AE, adverse event

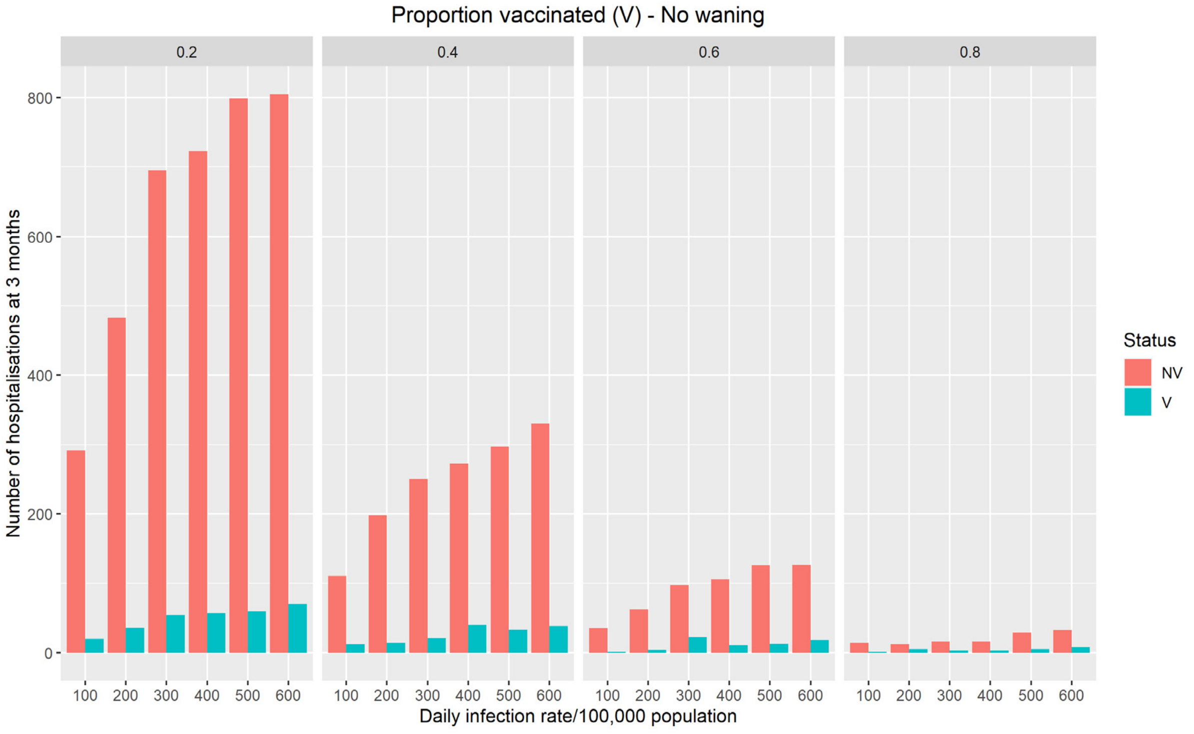

Figure S2: Expected daily number of hospitalisations at three months as a function of infection rate, vaccination status and vaccine coverage

NV, not vaccinated; V, vaccinated

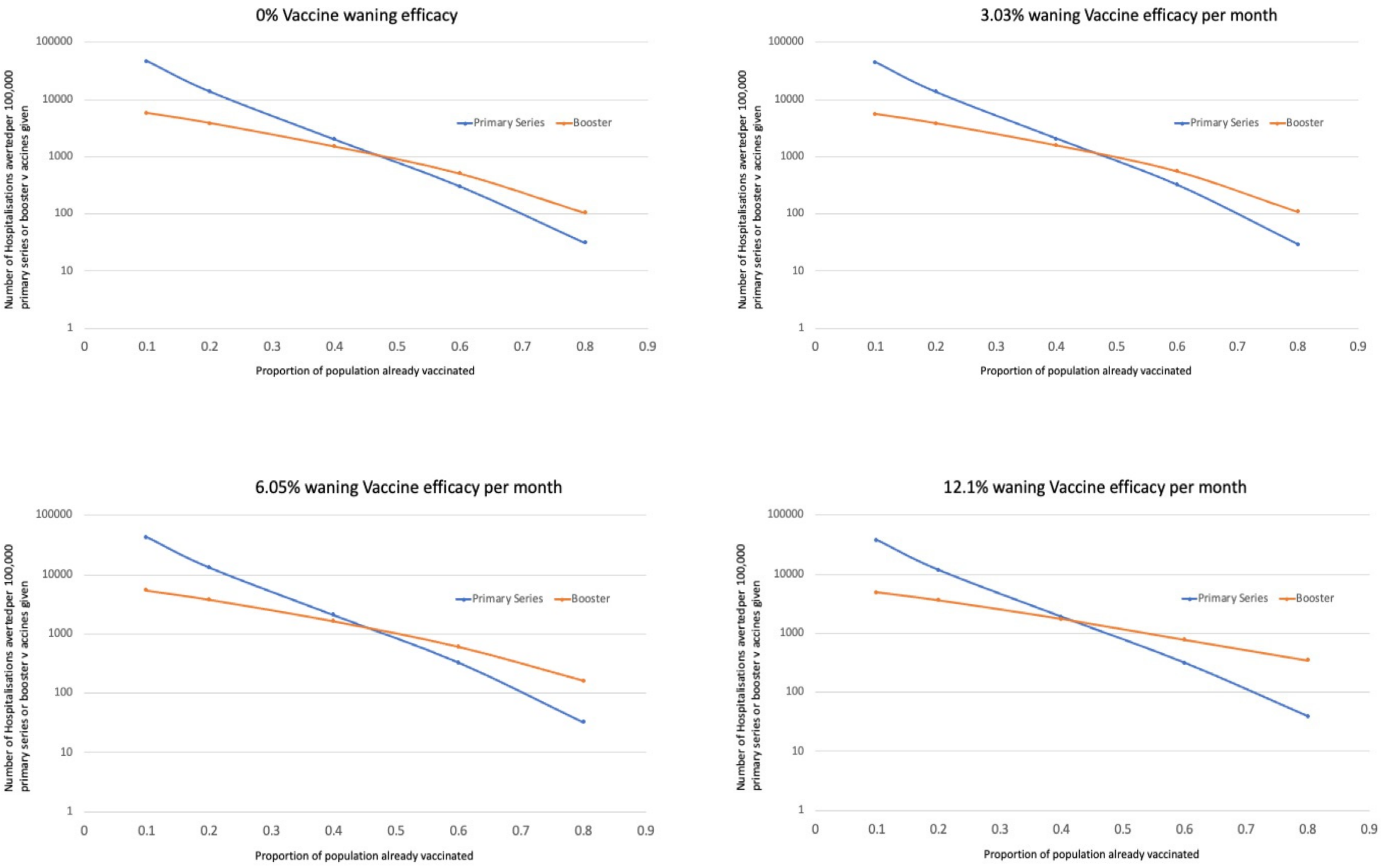

Figure S3: The number of COVID-19 hospitalisations prevented per 100,000 primary series or booster vaccines (logarithmic scale) for different levels of vaccination protection waning

REFERENCES

1. Shiri T, Evans M, Talarico CA, Morgan AR, Mussad M, Buck PO, et al. Vaccinating Adolescents and Children Significantly Reduces COVID-19 Morbidity and Mortality across All Ages: A Population-Based Modeling Study Using the UK as an Example. Vaccines (Basel). 2021;9(10).
2. Salje H, Tran Kiem C, Lefrancq N, Courtejoie N, Bosetti P, Paireau J, et al. Estimating the burden of SARS-CoV-2 in France. Science. 2020;369(6500):208-11.
3. Diekmann O, Heesterbeek JA, Metz JA. On the definition and the computation of the basic reproduction ratio R0 in models for infectious diseases in heterogeneous populations. J Math Biol. 1990;28(4):365-82.
4. Wölfel R, Corman VM, Guggemos W, Seilmaier M, Zange S, Müller MA, et al. Virological assessment of hospitalized patients with COVID-2019. Nature. 2020;581(7809):465-9.
5. Imai N, Cori A, Dorigatti I, Baguelin M, Donnelly CA, Riley S, et al. Transmissibility of 2019-nCoV. Imperial College London (25-01-2020). 2021. Available from: https://doi.org/10.25561/77148.
6. Shimabukuro T. COVID-19 Vaccine safety updates. CDC; 2021 June 23, 2021.
7. Office for National Statistics. Population estimates by output areas, electoral, health and other geographies, England and Wales: mid-20192020 19 July 2021. Available from: https://www.ons.gov.uk/peoplepopulationandcommunity/populationandmigration/populationestimates/bulletins/annualsmallareapopulationestimates/mid2019.
8. Mossong J, Hens N, Jit M, Beutels P, Auranen K, Mikolajczyk R, et al. Social Contacts and Mixing Patterns Relevant to the Spread of Infectious Diseases. PLOS Medicine. 2008;5(3):e74.
9. Ferguson N, Laydon D, Nedjati Gilani G, Imai N, Ainslie K, Baguelin M, et al. Report 9: Impact of non-pharmaceutical interventions (NPIs) to reduce COVID19 mortality and healthcare demand. 2020.
10. Smith C, Odd D, Harwood R, Ward J, Linney M, Clark M, et al. Deaths in Children and Young People in England following SARS-CoV-2 infection during the first pandemic year: a national study using linked mandatory child death reporting data. medRxiv. 2021:2021.07.07.21259779.
